# The Impact of Cavities in Different Thermal Applications of Nanofluids: A Review

**DOI:** 10.3390/nano13061131

**Published:** 2023-03-22

**Authors:** Mudasar Zafar, Hamzah Sakidin, Mikhail Sheremet, Iskandar Dzulkarnain, Roslinda Mohd Nazar, Abida Hussain, Zafar Said, Farkhanda Afzal, Abdullah Al-Yaari, Muhammad Saad Khan, Javed Akbar Khan

**Affiliations:** 1 Department of Fundamental and Applied Sciences, Universiti Teknologi PETRONAS, Bandar Seri Iskandar 32610, Malaysia; 2 Center for Research in Enhanced Oil Recovery, Universiti Teknologi PETRONAS, Bandar Seri Iskandar 32610, Malaysia; 3 Laboratory on Convective Heat and Mass Transfer, Tomsk State University, 634050 Tomsk, Russia; 4 Department of Petroleum Engineering, Universiti Teknologi PETRONAS, Bandar Seri Iskandar 32610, Malaysia; 5 Department of Mathematical Sciences, Faculty of Science & Technology, Universiti Kebangsaan Malaysia UKM, Bangi 43600, Malaysia; 6 Department of Sustainable and Renewable Energy Engineering, College of Engineering, University of Sharjah, Sharjah 27272, United Arab Emirates; 7 U.S.-Pakistan Center for Advanced Studies in Energy (USPCAS-E), National University of Sciences and Technology (NUST), Islamabad 44000, Pakistan; 8 Department of Industrial and Mechanical Engineering, Lebanese American University (LAU), Byblos P.O. Box 13-5053, Lebanon; 9 Department of Humanities and Basic Sciences, MCS, National University of Sciences and Technology (NUST), Islamabad 44000, Pakistan; 10 CO_2_ Research Center, Universiti Teknologi PETRONAS, Bandar Seri Iskandar 32610, Malaysia; 11 Department of Petroleum Engineering, Texas A&M University at Qatar, Doha 23874, Qatar; 12 Institute of Hydrocarbon Recovery, Universiti Teknologi PETRONAS, Bandar Seri Iskandar 32610, Malaysia

**Keywords:** cavities, microchannel heat exchangers, nanoparticles, solar collector, heat transfer, magnetic field

## Abstract

Nanofluids and nanotechnology are very important in enhancing heat transfer due to the thermal conductivity of their nanoparticles, which play a vital role in heat transfer applications. Researchers have used cavities filled with nanofluids for two decades to increase the heat-transfer rate. This review also highlights a variety of theoretical and experimentally measured cavities by exploring the following parameters: the significance of cavities in nanofluids, the effects of nanoparticle concentration and nanoparticle material, the influence of the inclination angle of cavities, heater and cooler effects, and magnetic field effects in cavities. The different shapes of the cavities have several advantages in multiple applications, e.g., L-shaped cavities used in the cooling systems of nuclear and chemical reactors and electronic components. Open cavities such as ellipsoidal, triangular, trapezoidal, and hexagonal are applied in electronic equipment cooling, building heating and cooling, and automotive applications. Appropriate cavity design conserves energy and produces attractive heat-transfer rates. Circular microchannel heat exchangers perform best. Despite the high performance of circular cavities in micro heat exchangers, square cavities have more applications. The use of nanofluids has been found to improve thermal performance in all the cavities studied. According to the experimental data, nanofluid use has been proven to be a dependable solution for enhancing thermal efficiency. To improve performance, it is suggested that research focus on different shapes of nanoparticles less than 10 nm with the same design of the cavities in microchannel heat exchangers and solar collectors.

## 1. Introduction

Fluid flow and heat transfer in cavities due to nanofluid transport are critical in many engineering applications, both theoretically and practically. Some examples are nuclear and chemical reactors, electronic device cooling, solar power collectors, and thin film solar energy collector devices [1,2,3,4,5,6,7,8].

In recent decades, natural convection enclosures have garnered a lot of attention due to the significance of these enclosures and the diverse range of applications in fields such as the engineering of furnaces and the cooling of electronic systems. Increasing the effectiveness of heat transfer within these systems is typically a topic of significant importance from the perspective of conserving energy. Over the past few decades, a lot of computational work has been performed to figure out what the main mechanism is that makes nanofluids better at transferring heat. Because of these things, many researchers have been looking at natural convection in rooms with different temperatures [9].

Regarding problems with nanofluids, many researchers worldwide have been exploring the idea of modifying their cavities, for instance, a square cavity by Mansour et al. [10] and Mansour and Ahmed [11], while Azizul et al. [12] have investigated the heat transfer in a wavy cavity. Performance is also affected by thermo-fluid system parameters such as the heat transfer fluid properties, flow regimes, and geometrical parameters. Even though many of the procedures for vented cavity applications solve two-dimensional configurations, the more realistic approach is to use three-dimensional models. The improvement of heat transfer can be accomplished in a variety of different ways. These techniques change the flow patterns in the systems and the way heat moves through them. Selimefendigil and Oztop [13] have used a rotating cone-shaped object and carbon nanotube particles in the base fluid to study how thermoelectric power can be made in cavities with ventilation holes. The effect of the shape of the cavities on the TEG energy conservation of nanofluids is examined by Selimefendigil et al. [14]. Rectangular, triangular, L-shaped, and U-shaped cavities were studied, while alumina-water nanofluid performance increased. The results indicated that power rises to 87.8% with a higher opening ratio of the cavities.

The thermal performance of several cavity receiver shapes has been investigated experimentally, as described in [15]. Loni et al. [16] have investigated two oil-based nanofluids in a hemispherical cavity receiver and discovered that an alumina/oil nanofluid in a hemispherical cavity receiver is the best choice in terms of energy and exergy. Other scientists have created a new two-step dish concentrator with a cavity receiver to heat air for use as the working fluid in a turbine. With the development of micro-machining technology, improving the structure of microchannel heat sinks has become an excellent way to improve how well they transfer heat. Optimization of turbulence structures is carried out mainly by changing the cross-section shape of the microchannel [17]. The microchannel cooling technique with nanofluid is one of the valuable measures for high-power-density devices with limited area and rapid heat dissipation. Li et al. [18] have investigated the heat transfer enhancement mechanism of nanofluids in a microchannel with non-uniform cavities in terms of the coupling effect of thermal boundary layer thickness, perturbation, and axial thermal conduction. Researchers [19,20,21,22] have used nanofluid transport cavities for an oil recovery application.

**Figure 1:**
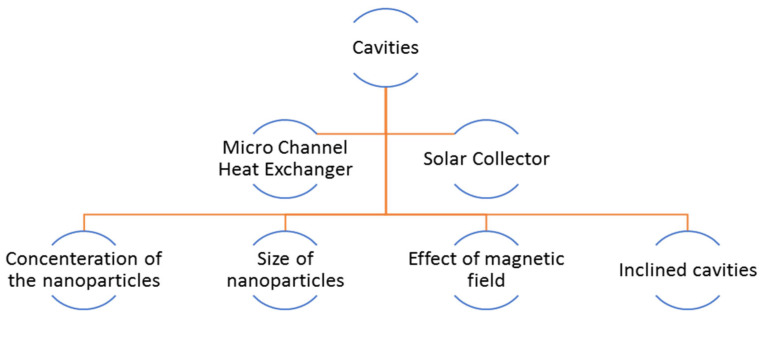
Flow chart of the review.

The literature mentioned above shows that cavities are essential in industrial and real-world applications such as solar collectors, heat exchangers, cooling technology, and civil technology. Figure 1 describes the flow chart of this review.

In the next section we will discuss some important mathematical models investigated by researchers to study the heat-flow problems using different geometries.

### 1.1. Buongiorno Nanofluid Model

Some years ago, Buongiorno [23] presented a mathematical model for the description of convective heat transport in nanofluids with the goal of exploring the influence of several slip mechanisms in nanofluids. The model addresses the heat-transfer rate in two ways: laminar sublayers and a reduction in velocity. In addition, the study focuses on seven slip mechanisms. The results demonstrate that the effects of Brownian diffusion and thermophoresis are critical slip mechanisms in nanofluids.

Non-dimensional analysis of the equations reveals that energy transfer by nanoparticle dispersion is insignificant, and thus the unusual heat-transfer rate increases cannot be demonstrated. In addition, a comparison of the nanoparticle and turbulent eddy time and length scales shows that the nanoparticles move uniformly with the fluid in the existence of turbulent eddies. Hence, an influence on turbulence intensity is also speculative. As a result, the model provides an alternative justification for the unusual heat-transfer rate rises: the nanofluid properties may vary considerably within the boundary layer due to the temperature gradient and thermophoresis impact. These consequences can cause a significant reduction in viscosity within the boundary layer of the heated fluid, resulting in improved heat transfer.

There is the following hypothesis to construct the model:

1. The flow is incompressible;
2. There is no chemical reaction between them;
3. The effect of external force is neglected;
4. The dilute mixture is less than one;
5. There is no effect of viscous dissipation;
6. No consideration is given to radiative heat transfer;
7. Local thermal equilibrium between nanoparticles and clear fluid.

The governing equations based on the above assumptions are:

(1) ∇.v=0

(2) $$\frac{\partial \varphi }{\partial t}+v.\;\nabla \varphi ={\;D}_{B}\nabla^{2}\varphi +D_{T}\;\frac{\nabla^{2}T}{T}$$

(3) $$\rho \left[\;\frac{\partial v}{\partial t}+v\;.\;\nabla v\;\right]=-\nabla p-\nabla \;.\;\tau$$

(4) $$\rho c\left[\;\frac{\partial T}{\partial t}+v.\nabla T\;\right]=\;k\nabla^{2}T+{\;\rho }_{P}c_{p}\;\left[D_{B}\nabla \varphi \;.\nabla T+{\;D}_{T}\frac{\nabla^{2}T\;}{T}\;\right]$$

(5) $$D_{B}=\frac{k_{B}T}{3{\mu d}_{P}}$$

(6) $$D_{T}=\frac{\beta \mu \varphi }{\rho }$$

(7) $$\tau =-\;\mu \;[\;\nabla v+{\left(\nabla v\right)}^{t}]$$

It is worth noting that the conservation equations are tightly coupled such that velocity is influenced by viscosity; the temperature of the nanofluid is controlled by thermophoresis and Brownian diffusion; temperature is caused by thermal conductivity as well as the Brownian and thermophoretic terms in the energy equation; and the temperature of the nanofluid is affected by the velocity of the nanofluid because of the convection terms in the nanoparticle continuity and energy equations, respectively. The second and third terms on the right-hand side of Equation (4) can be ignored when solving nanofluid heat-transfer problems, yielding the energy equation for a nanofluid explicitly identical to that of a pure fluid. It is also found that energy transfer by nanoparticle dissipation is inconsequential compared to heat conduction and convection.

### 1.2. Tiwari and Das Nanofluid Model

Tiwari and Das [24] developed a single-phase nanofluid mathematical model to investigate the behavior of nanofluids in a square cavity driven by a differentially-heated two-sided cavity. Three different scenarios were examined. In one case, the left (cold) wall rises while the right (hot) wall falls. In instances II and III, the left wall moves downward while the right wall moves upward, and both walls move upward. In all three cases, the moving walls have the same speed, and the gravitational force is directed parallel to the moving walls. The following stages were taken in the fluid flow:

- The fluid is laminar, Newtonian, incompressible, and unsteady.
- The nanoparticles are considered to have a homogeneous shape and size.
- The fluid phase and nanoparticles are thermally balanced and flow at the same rate.
- In comparison to other types of heat transmission, radiation heat transfer between sides is insignificant.
- Excluding for the density changes in the buoyancy force, which is dependent on the Boussinesq approximation, the thermophysical parameters of the nanofluid are considered to remain constant.

Following are the equations that comprise the mathematical model:

(8) ∇.v=0

(9) $$\frac{\partial u}{\partial t}+\frac{\partial u^{2}}{\partial x}+\frac{\partial u.v}{\partial y}=-\frac{1}{\rho_{\mathrm{nf},0}}\;\nabla p+\frac{\mu_{\mathrm{eff}}}{\rho_{\mathrm{nf},0}}\;\nabla^{2}u$$

(10) $$\frac{\partial v}{\partial t}+\frac{\partial u.v}{\partial x}+\frac{\partial v^{2}}{\partial y}=-\frac{1}{\rho_{\mathrm{nf},0}}\;\nabla p+\frac{\mu_{\mathrm{eff}}}{\rho_{\mathrm{nf},0}}\;\left(\nabla^{2}v\right)+\frac{1}{\rho_{\mathrm{nf},0}}({\varphi \rho }_{s,0}\beta_{s}+\left(1-\varphi \right){\;\rho }_{f,0}\beta_{f}\left(T-{\;T}_{C}\right)$$

(11) $$\frac{\partial T}{\partial t}+\frac{\partial u.T}{\partial x}+\frac{\partial v.T}{\partial y}=\alpha_{\mathrm{nf}}\left(\nabla^{2}T\right)$$

(12) $$\alpha_{\mathrm{nf}}=\frac{k_{\mathrm{eff}}}{{\left({\rho C}_{P}\right)}_{\mathrm{nf},0}}$$

In the next section, we will briefly discuss the effect of cavities in nanofluid transport and different parameters such as the effect of nanoparticles, the effect of the concentration of the nanoparticles, the effect of the magnetic field, and the effect of the heaters and coolers in the cavity for heat-transfer enhancement. In Section 2, we will discuss the role of cavities in microchannel heat exchangers and in Section 3, we will briefly discuss the importance of the cavities in solar collectors.

## 2. The Role of Cavities in Nanofluid Transport

**Figure 2:**
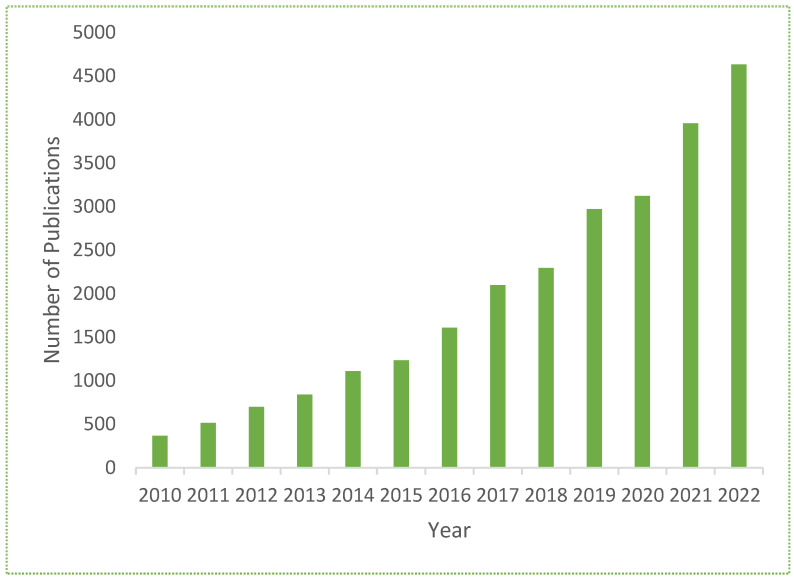
Published articles on nanofluids (Source: Scopus database, 2010–2022).

Nanofluids and nanotechnology are very important in enhancing heat transfer due to the thermal conductivity of their nanoparticles, which play a vital role in heat transfer applications. Nanofluids are engineered colloidal suspensions with different small particles of sizes ranging from 1 nm to 100 nm in pure fluids (clear fluids), such as water, ethylene glycol, and engine oil. Figure 2 provides a graphical picture of the published articles in the field of nanofluid flow.

For the last twenty years, researchers have used the concept of cavities/geometers in nanofluid transport to obtain maximum heat-transfer enhancement. Figure 2 describes the importance of nanofluids in different heat-transfer applications and the number of published research articles showing the researchers’ attractions in this domain. Table 1 lists the numerous cavities utilized in nanofluids that various researchers have theoretically and quantitatively studied.

It also emphasizes the significance of cavities in nanofluids for improving heat transfer in multiple applications. In this paragraph, we explain the use of cavities in nanofluid flow, as shown in Table 1.

Sheikholeslami and Vajravelu [25] investigated the outcome of a fluctuating magnetic field influence on the flow of hydrothermal nanofluids in a square cavity heated from the bottom. Armaghani et al. [26] explored the behavior of an L-shaped cavity with the mixed convective and nanofluid flow. In the existence of an inclined magnetic field, Rahman et al. [27] investigated free convection heat transfer in an isosceles triangular cavity filled with nanofluid. Using a closed elbow-shaped cavity, Ebrahimi et al. [28] examined the nanofluid transport behaviors in heat transfer. Miroshnichenko et al. [29] studied the impact of radiation on alumina–water-based nanofluid flow in a shallow cavity with a nanoparticle size of 10 nm. Safaei et al. [30] investigated free convection in an open trapezoidal cavity filled with a copper-based water nanofluid. Sheremet et al. [31,32,33,35] investigated the impact of wavy wall cavities, porous open cavities, and inclined wavy cavities filled with nanofluids on flow structures and heat transfer. Sheikholeslami et al. [36] investigated the effect of a uniformly inclined magnetic field in a vented hollow with an elastic step-like wall groove on fluid flow and energy transport, which is predicted to produce several separation zones. Santra et al. [37] examined the impact of a copper–water nanofluid in a square differentially heated cavity, treating the nanofluid as non-Newtonian in nature. Hwang et al. [38] explored the phenomenon of natural unstable convection and the heat transmission attributes of a water-based Al_2_O_3_ nanofluid in a rectangular enclosure heated from below. Solomon et al. [39,40] investigated the effect of the cavity aspect ratio on natural convection. Iachachene et al. [41] conducted a numerical study on melting paraffin wax embedded in a trapezoidal cavity using the enthalpy–porosity technique. Bairi [42] investigated the nanofluid transport in a porous hemisphere filled with a ZnO–H_2_O nanofluid. Heris et al. [43] thoroughly investigated the impact of using three different turbine oil-based nanofluids in a cubic cavity with Al_2_O_3_, TiO_2_, and CuO nanoparticles on flow structures and energy transfer.

**Figure 3:**
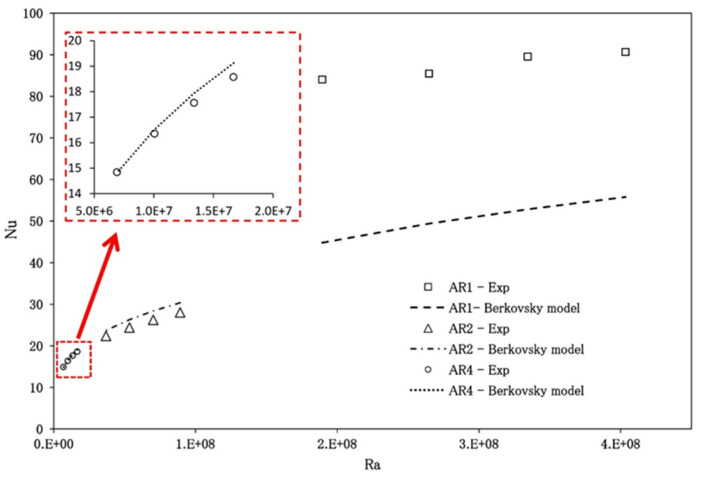
Impact of different aspect ratios on cavities [39].

According to the literature, appropriate cavity design is essential in thermal systems. Using L-shaped cavities is hugely beneficial in the cooling systems of nuclear and chemical reactors and electronic components [26]. In solar thermal collectors, an isosceles triangular cavity has practical applications [27]. Given the reputation of experimental works, better design, and industrial necessities for rectangular cavities, the effect of a cavity’s aspect ratio on the heat transfer coefficient and Nusselt number is significant. Researchers have worked on different sizes of cavity and its aspect ratio to find the proper design of cavities for the maximum possible results in relevant applications. Figure 3 depicts the aspect ratio phenomenon in cavities.

Because of the buoyancy effect’s varying intensity, an increase in the cavity aspect ratio causes an increase in the rate of heat transfer. A decrease in the size of the aspect ratio results in an increase in the size of the cavity as well as the charge quantity, which ultimately results in increased buoyancy as well as heat transfer. Additionally, an increase in the aspect ratio of the cavity causes a rise in the heat-transfer area, significant frictional losses, and a decrease in the flow intensity. All of these effects are a direct result of the increase in the cavity’s size. When compared to the high aspect ratio, the friction losses are reduced when the cavity has a lower aspect ratio, which causes the Rayleigh number (Ra) to increase. This leads to improved natural convection becoming the dominant flow pattern in the cavity. This contributes to a greater amount of heat transfer within the cavity.

One can notice that the heat-transfer abilities of the cavities rise when the temperature difference increases. The reason for this is that as the temperature difference increases, so does the buoyancy effect inside the cavity, which aids in heat transfer, increases fluid circulation, and reduces the density of the fluid inside the cavity. As a result, buoyancy forces grow, fluids move upwards very quickly, and fluid circulation increases, resulting in maximum heat transfer [39,40]. The thermophoresis effect that happens inside the cavity is the main reason why the rate of heat transfer speeds up in cavities when the temperature difference becomes bigger.

This section concludes with the following summary of the application of cavities in nanofluid transport. Using cavities in nanofluid transport increases the heat-transfer rate, and the cavities’ design and dimensions are crucial in various industrial applications. In the literature, several instances of the usefulness of different cavities in multiple applications, such as L-shaped cavities used in the cooling system of nuclear and chemical reactors and electronic components, are examined. Open cavities such as ellipsoidal, triangular, trapezoidal, and hexagonal are applied in electronic equipment cooling, building heating and cooling, and automotive applications. A triangular isosceles cavity has applications in solar thermal collectors. Consequently, cavity design is a crucial part of thermal systems. Appropriate cavity design conserves a substantial amount of energy; hence, suitable cavity design in relevant applications produces favorable rates for heat transfer.

In the next section, we will discuss the effect of various parameters, such as nanoparticle concentration, magnetic field effect, nanoparticle effect, inclined cavities, solid and circular cylinders, and the impact of the heater and cooler on the cavities.

### 2.1. Effect of the Nanoparticle Concentration in Cavities

Table 1 and Table 2 show that the volume concentration of nanoparticles also plays a significant role in cavities for heat-transfer enhancement. Researchers most commonly study volume concentrations between 0.01 and 0.08. As the volume concentration increases, the average Nusselt number rises too and as a result the heat-transfer rate inside the cavity increases. Still, it gradually decreases as the volume concentration increases from 0.05% because, at this point, a larger volume concentration increases the density and viscosity of the nanofluids and for this effect of the buoyancy forces becomes weak, i.e., the buoyancy force decreases. As a result, minimal movement occurs in the fluid, which slows down the fluid circulation and, as a result, heat transfer [25,34,35,36,39,40]. The effect of the concentration of nanoparticles on the cavity heat-transfer rate is illustrated in [40].

As the buoyant force increases, the mode of heat transmission shifts from conduction to convection, as observed by Sheikholeslami and Vajravelu [25]. In the absence and presence of a magnetic field, nanoparticle addition to the base fluid increases the heat transfer by an average of 9–9.5%. The addition of nanoparticles suppresses convective flow while increasing the average Nusselt number [34]. When Ra = $10^{4}$ and Φ = 0.3%, adding hybrid nanoparticles maximises the Nusselt number [35]. The optimal nanoparticle concentration is significantly influenced by the aspect ratio (AR). The optimal nanoparticle concentration for maximal heat transmission increases as AR rises. These findings are useful for the design of cavities employing nanofluids as working fluids in applications such as solar collectors and heat exchangers [36]. A nanofluid with a nanoparticle volume concentration of 0.05% is optimal for maximum heat transfer since it transfers 10% more heat than the base fluid in the porous cavity [25,34,35,36,39,40].

The effect negatively impacts heat-transfer performance on the heat-transfer coefficient and temperature in the cavity’s cold wall. The main explanation is that the nanoparticles switch from hot to cold as the temperature rises. Since more nanofluid is close to the cavity’s chilly walls, a thin coating forms. Small quantities of fluid fill the top of the cavity due to the buoyancy of the fluids, whereas large concentrations fill the bottom—the fluids in the top and bottom areas of the cavity move as a result. The Nusselt numbers are lower because this effect is more severe in the cold wall than in the hot wall. It indicates that the temperature on the cavity’s cold side considerably impacts the Nusselt numbers and heat-transfer efficiency in cavities.

In the end, the effect of concentration summarizes that the effective concentration of nanoparticles and the temperature gradient significantly impact the cavities’ heat transfer capabilities. Cavities’ highest heat-transfer rate is attainable between 0.03 and 0.05%. The heat-transfer rate falls if we increase the volume concentrations outside this range.

### 2.2. Effect of the Nanoparticles in Cavities

The heat-transfer rate inside the cavity increases when the nanoparticles add to the base fluids. Therefore, nanoparticle size is crucial for achieving the highest heat-transfer rate. According to the literature, a size of nanoparticles between 10–50 nm is more stable in base fluids in cavities at the specified temperature gradient [29]. The reason for this is that the effective thermal conductivity of nanofluids begins to decrease with the increment in size of nanoparticles if we increase the size of the nanoparticles beyond the range described above. After that, the viscous force of the nanofluids within the cavity begins to decrease, which results in a reduction in buoyancy as well as the rate of heat transfer.

Table 2 shows further information about the size of nanoparticles studied to examine heat-transfer processes in cavities. The highest heat-transfer enhancement comes when the nanoparticle size decreases and the average temperature of the fluid increases [60,61]. Authors have demonstrated the effect of nanoparticles sizes: $d_{p}$ = 25 nm, 85 nm, and 145 nm were investigated in a square cavity for maximum heat-transfer rate and the heat-transfer rate was maximal at $d_{p}$ = 25 nm and insignificant for particles larger than 85 nm in diameter. Garoosi et al. [61] studied three different types of the effect of nanoparticles having ranges of $25\;\mathrm{nm}\leq d_{p}\leq 145\;\mathrm{nm}$ on the heat-transfer rate and concluded that when the diameter of the nanoparticle is reduced, more heat transfer is achieved. It was also noted that when the values of the Rayleigh number were high along with a smaller diameter of nanoparticles more heat transfer was achieved.

Taking into account the research of Azimikivi et al. [63], the most significant heat-transfer rate comes if the diameter of the nanoparticles is smaller ($d_{p}=50\;\mathrm{nm}$). Wang et al. [64] discovered the maximum heat-transfer rate for the diameter $d_{p}=25\;\mathrm{nm}$. The greatest heat transmission may be obtained by making the diameter of the nanoparticles smaller in the cavity. It is recommended that a smaller size of nanoparticles can enhance the heat transfer in cavities.

### 2.3. Effect of the Cavities’ Inclination Angle

The experts [44,72] have studied the inclined cavity’s mechanism to investigate heat transfer in nanofluids. The angle of inclination of a square cavity has a range from 0° to 60°. The heat-transfer behavior depends on the values of the nanoparticle volume fraction, and the heat-transfer rate can be either maximum or minimum by increasing or decreasing the importance of the nanoparticle volume fraction, according to the studies.

It specifies that for low Ra values and increasing inclination angle, maximal heat transmission comes at all nanoparticle volume fractions (φ=0.01−0.04). In addition to this, when the inclination angle is 0° and the lowest value of nanoparticle volume fraction is used, the heat-transfer rate is enhanced by 8%, but when the inclination angle increases to  60°, the heat-transfer rate increases to 24%. The effect of the rotation angle on the heat-transfer enhancement is illustrated in [75]. A cavity with an angle of 0° has the worst thermal performance, followed by a cavity with an angle of 45° and a cavity with an angle of 90°. A cavity whose inclination angle is 135° provides the lowest thermal performance of all the cavities [75]. When the inclination angle is equal to zero degrees, the side that is hot is at the bottom, and the side that is cold is at the top. Fluid closer to the bottom of the container heats up and moves upward, while fluid closer to the top of the container cools off and moves downward. Natural convection should also not be made more substantial for another reason, which is that hot and cold fluids move in opposite directions when they move. When cavities are involved, angles between 0 and 90° give the best improvement in heat transfer.

The inclination angle of the cavity and periodic thermal boundary conditions are two potential control parameters that are suitable for regulating the flow of heat and fluid inside the cavity. An increase in the angle of inclination is indicative of a non-linear influence on the rates of heat transfer and fluid flow. The heater effect of the temperature-varying wall produces the greatest increase in Nusselt number, which can be enhanced with an increase in the inclination angle from 0 to 90°. Any further increase will result in a lower value for this maximum as well as a shift in the position of the Nusselt numbers of the profiles. It seems to show that the angle of inclination of the cavity and the periodic thermal boundary conditions could be used to control the flow of heat and fluid inside the cavity.

### 2.4. Effect of the Heater and Cooler Inside Cavities

The impact of the heater and better locations inside the cavity is very significant for heat-transfer enhancement. These applications of nanofluids can be seen in heat exchangers. Authors [47,52] have investigated the effect of heater location and its arrangements on heat transfer. They determined that the heater should be placed entirely on the cavity’s left wall from a heat-transmission perspective. Garoosi et al. [60] explained the mechanism of the location of the heater in Nu at different values of Ra. Researchers showed that the average Nu is the highest because the nanofluid is heated near the heater and expands as it goes upwards. Conduction heat transfer is considerable around the cavity at low Ra values [60].

The intensity of the flow rate increases, and a small eddy forms around the cavity, both of which indicate that convection is occurring; additionally, increasing the values of the effective viscosity of the nanofluid results in a reduction in the maximum stream function, and changing the locations of the heater and cooler results in an exceptionally high ${\mathrm{Nu}}_{\mathrm{total}}^{-}$.

It was also discovered that because conduction is dominant at low Ra values, nanoparticles impede heat transmission at all heater positions. Therefore, placing the heater and cooler close to each other increases the heat-transfer rate because the conduction opposition decreases as the distance between them decreases, resulting in increased heat transfer. Another aspect where their location differs is that the hot and cold fluid around the heater and cooler flows quickly above and downward, improving heat transmission [60].

It was discovered that switching the positions of the heater and cooler along two neighboring straight sides of a quadrilateral cavity did not affect the flow direction in any way. The formation of a single circulation vortex is ultimately governed by the sidewall, regardless of whether the overall flow velocity has increased. This indicates that the production of thermal entropy is the fundamental driver of thermodynamic irreversibility [76]. Both the heater and cooler might have been positioned in either the second or third quadrant to provide the best possible heat transmission. The Lorentz force is an essential component in the complex system that is responsible for regulating the rate of heat transmission.

### 2.5. Effect of the Magnetic Field in Cavities

A great deal of theoretical research has been conducted on magnetohydrodynamics (MHD) and the natural convection of nanofluids using a variety of cavities. In the beginning, MHD was utilized to solve difficulties in geophysics and astrophysics. Since then, it has caught the attention of scholars in many different fields.

A review of these studies discovered that various heat transfer mechanisms, the ability to focus boundary conditions, cavity inclination angles, thermal distribution models, heater types, implantation of partitions, different configurations of objects in the cavities, different nanofluid types, porous media, different modelling methods, flow conditions, and behavior, and various magnetic field types, orientation, and inclination were used to investigate hydrothermal behavior. of The numerical work that has already been performed on the hydromagnetic behavior of nanofluids in various cavity shapes is summarized in Table 3. This table is a part of the larger work that has been conducted on this topic. The type of nanoparticles, their size, the range of Hartman numbers, the angle of the cavities’ inclination, the angle of the magnetic field, as well as a diagram, and the results of experimental studies are all included here. [16] investigates how the presence of a variable magnetic field affects the flow of hydrothermal nanofluids within a square cavity that is heated from below. Lorentz forces influence the flow of the nanofluid whenever a magnetic field is applied to a cavity. When there is a greater amount of Lorentz force, the axial velocity decreases, which results in an increase in the thickness of the temperature boundary layer. In addition, an increase in the Lorentz force causes the center of the primary eddy to move downward in response. The existence of a magnetic field also brings about a reduction in the amount of isothermal distortion that occurs. When nanoparticles of Fe_3_O_4_ are introduced into the mixture, there is an increase in the temperature gradient. This increase can be seen to be more noticeable when there is also a magnetic field within the vicinity. When the system is operating in a conduction mode, the addition of Fe_3_O_4_ has a significant impact on the thermal conductivity of the system. Therefore, an increase in the Lorentz force results in an increase in the rate of heat transfer. However, the opposite is true when it comes to the buoyancy force; an increase in the buoyancy force results in a decrease in the rate of heat transfer. It indicates that the impact of adding nanoparticles to a base fluid will decrease as the strength of the convective heat transfer increases. This is because the convective heat transfer will become stronger as time goes on.

In [70], the influence of an Al_2_O_3_–water nanofluid on laminar natural convection heat transfer was investigated while a magnetic field was present in an open cavity. The heat-transfer mechanism under study was natural convection. It causes the heat transfer to become more efficient by increasing the volume fraction in several different Hartmann numbers, which in turn causes the heat transfer to become more effective. In addition, it makes perfect sense that nanoparticles would have a further significant influence on the isotherm at the value of 104 in the direction of pure fluid at the value of 0. The isotherms of the nanofluid and the fluid perfectly overlap each other at the open boundary, but as they move closer to the hot wall, they start to move further and further apart from each other. Because of this, the effect of the nanoparticle is negligible at the open boundary partition; however, the effect gradually increases as the fluid moves inside the enclosure. In addition, an increase in the Hartmann number causes a decrease in the maximum value of the stream function, while the rate at which this occurs varies depending on the Rayleigh number. This is because the maximum value of the stream function is proportional to the Hartmann number. As an illustration, when going from Ha = 0 to 30, the values of the maximum stream function decrease by 63%, 41%, and 29%, respectively. The Rayleigh numbers Ra = 10^4^, 10^5^, and 10^6^ correspond, respectively, to these percentages. Because of this, the influence of the magnetic field on the circulation of the fluid loses some of its significance as the Rayleigh number increases. The effect of nanoparticles on streamlines becomes abundantly clear when the values of the maximum stream function and the motivation of streamlines both increase for varying Hartmann and Rayleigh numbers. As a direct consequence of this, the presence of nanoparticles in an open enclosure has the effect of enhancing the buoyancy-driven circulations within the space. In addition, nanoparticles of varying Hartmann and Rayleigh numbers exhibit a wide range of behaviors on the streamlines due to the erratic manner in which they raise the maximum stream function.

Researchers are looking at how a tilted uniform magnetic field affects the natural convective flow of fluids and heat transfer in a trapezoidal cavity with a hot inclined wall that is open on one side. This effect is being looked at by researchers from the perspective of a trapezoidal cavity [20]. In the situation where there is no magnetic field present (Ha = 0), it has been found that a formation of intense circulation can take place inside a cavity that is only partially open. This was discovered in a scenario where there is no magnetic field present. In this scenario, nanofluid enters the open boundary on the right through the lower part of the boundary, and it exits the same boundary through the upper part of the boundary. The application of an external magnetic field that is consistent throughout its entirety is what ultimately leads to a reduction in the amount of flow and heat transfer that occurs within the cavity. In the situation in which Ha equals 50 and the magnetic field is oriented horizontally (equals 0), the Lorentz force will be in a state of parallelism with the gravitational force. This will be the case because the magnetic field will be aligned horizontally. The appearance of a magnetic field is responsible for a slowing of the motion of the velocity boundary layer and an increase in the thickness of the thermal boundary layer. This is because the magnetic field causes the cooling that comes from the open boundary to be of a lower intensity and therefore enter the cavity. While this is happening, the ascending flow sizes increase as they become closer to the inclined wall. A rotation of the magnetic field through an angle of π/4 causes a significant change in the internal circulation, resulting in the formation of a vortex within the cavity. This change will manifest itself as a significant change to the internal circulation. Because of the presence of this low-intensity vortex, which is situated near the hot wall, the size of the ascending flow has been significantly reduced. At the same time, the temperature distribution reveals a predominance of a conductive heat transfer mechanism, with isotherms that are quasi-parallel to the boundaries of hot and cold regions. This indicates that the heat is being transferred from the hot region to the cold region. When the Lorentz force and the gravity force are aligned perpendicular to one another (π/2), the condition known as the parallelogram state exists. In a study, a more significant suppression of the convective flow and heat transfer was achieved through the generation of a central vortex, which stopped the heat pickup from the inclined wall. This was accomplished by preventing the heat from being absorbed by the wall. In this instance, the cooling of the cavity does not make a significant contribution, and the isotherms also repeat the forms of the heater and the cooler. It is essential to keep in mind that the recently formed central vortex has the effect of diminishing the intensity of the incoming cold flow from the surrounding areas. This is something that should always be kept in mind. The subsequent rotation of the magnetic field, which is represented by the equation 3π/4, causes an intensification of the internal circulation, which in turn causes a displacement of the central vortex to the open boundary and an essential widening of the upward flow near the inclined hot wall. This cycle continues until the internal circulation reaches its maximum intensity and the central vortex reaches its new location. In places where heat conduction is the most common way for heat to move, increasing the strength of the magnetic field makes the convective flow and heat transfer slow down even more.

The consequence of a magnetic field on nanofluid transportation in an open porous cavity is something that researchers are investigating [69]. The effects of the Darcy, Rayleigh, and Hartmann numbers, as well as the volume fraction of CuO, on the behavior of hydrothermal systems are being studied. The thickness of the thermal boundary layer increases when CuO nanoparticles are added to the mixture. In situations with a low permeability, this increase can be seen more clearly. In situations such as these, an increase in the Lorentz forces causes a greater number of changes because of the addition of nanoparticles. When nanoparticles are introduced into a nanofluid, the motion of the nanofluid increases. This, in turn, results in an improvement in the flow circulation and the transport of thermal energy through the nanofluid. The application of the magnetic field effect causes a decrease in the size of the upper eddy, and the Lorentz forces work toward lowering the amount of isothermal distortion that takes place as a result.

Chandra et al. [71] investigated the fluid flow and heat-transfer characteristics of nanofluid while it is contained in a U-shaped cavity and subjected to a magnetic field. The ${\mathrm{Nu}}_{\mathrm{avg}}$ numbers rise with increasing AR (aspect ratio of the cavity) and *n* (power law index). When looking at the set of results for AR = 0.2, it is quite clear that the magnetic inclination angle has a nearly insignificant effect on the ${\mathrm{Nu}}_{\mathrm{avg}}$ when the value of *n* is equal to 0.6. The average increases linearly with boosting for *n* = 1 and 1.4, and it reaches its maximum value somewhere around 30°. As soon as it reaches its maximum value, the average Nu immediately starts to decrease in a linear fashion. When AR equals 0.4, the effect of the magnetic inclination angle on ${\mathrm{Nu}}_{\mathrm{avg}}$ begins to decrease. This happens because AR is beginning to cancel out the effect. The value of Nu avg will increase when AR is set to 0.6, but the rate of increase will slow down after 60°. This is because the value of ${\mathrm{Nu}}_{\mathrm{avg}}$ increases as the angle increases.

Using Fe_3_O_4_ nanoparticles, researchers [72] investigate how the influence of Lorentz forces will affect ferrofluid-free convection when there is thermal radiation present in a tilted cavity. This is tested in order to determine how the influence of Lorentz forces will affect ferrofluid-free convection. The rate of heat transfer is positively influenced by the effect that the radiation parameter has on the nanofluid. This effect’s significance is heightened when a magnetic field is present in the system. Ghalambaz et al. [73] studied the effect of a changing magnetic field on the heat transfer of a nanofluid made of Fe_3_O_4_ and water. They carried out their work in a half annulus cavity.

A lattice Boltzmann method is used to model MHD Cu–water nanofluid flow in a cavity [74]. Four nanoparticle volume fractions (φ = 0, 0.02, 0.04, and 0.06), three Rayleigh numbers (Ra = 10^3^, 10^4^, and 10^5^), a wide range of Rayleigh Hartmann numbers (Ha = 0, 20, 40, and 60), and dimensionless heat generation or absorption (q = 10, 5, 0, 5, and 10) were computed. When the Hartmann number increases (Ha = 20, 40, and 60), the Lorentz force, which is generated as a result of the magnetic field effect, becomes higher than the buoyancy force. This is because the magnetic field effect generates the Lorentz force. This results in a reduction in the intensity of the flow circulation, which, in turn, causes the convection effect to start becoming less effective. As the Hartmann number increases, the cell center will move deeper and deeper into the cavity until it reaches its lowest point. This will continue until the cavity has reached its maximum depth.

In conjunction with this development, the length of the circulation cells starts to increase in an upward and vertical direction. Changes in the Rayleigh and Hartmann numbers are also known to influence the isotherms. At a Ra value of 10^3^, the effect of the Hartmann number on the isotherms is more pronounced than it is at an Ra value of 10^4^, which is the point at which the disparity between the two compared isotherms begins to grow noticeably. At a Reynolds number of 105, when the power of the convective flow begins to increase, the effect of the convective heat transfer begins to become more significant, and distinct boundary layers begin to form along the active wall of the cavity. These changes take place in the cavity. Because heat moves in this way, convective heat transfer is the most common way for heat to move from one place to another. In addition, the convection effects become more pronounced as the Rayleigh number increases, which causes the isotherms to become more warped. This occurs because the Rayleigh number is increasing. An increase in the Hartmann number on a global scale causes an increase in the Lorenz force, which ultimately results in a significant reduction in the convection that is occurring. When a magnetic field is applied, the temperature intensities of the fluid that is contained within the cavity decrease. This phenomenon is most pronounced at higher Rayleigh numbers.

A numerical investigation of the MHD effect linked with a fluid–structure interaction model was carried out in the context of a nanofluid-filled lid-driven cavity problem [76]. This was carried out for the case of volumetric internal heat generation, and it covered a wide range of flow parameters. Numerical analysis was performed on the Richardson number (0.01 ≤ Ri ≤ 100), the internal Rayleigh number ($10^{3}$–$10^{6}$), the Hartmann number (0–50), the inclination angle of the magnetic field (0°−90°), Young’s modulus of the flexible wall (5 $\times 10^{2}$–$10^{6}$), and the nanoparticle volume fraction (φ = 0–0.05) to determine how the values of these variables impacted flow and heat transfer. The cavity was filled with a CuO–water nanofluid via a magnetic field, which also generates volumetric heat. When the Richardson number is low, it is easier to observe mechanically powered left wall forced convection than it is to observe natural convection. When Ri = 0.01, the moving lid causes the flexible wall to concave inward. A value of Ri = 1 produces a primary cavity vortex as well as a secondary recirculation zone close to the heated wall. Convection, both strong and natural, is increased when Ri = 100 is applied.

The effect of buoyancy causes the flexible wall to become convex. Internal heating is more affected by the Richardson number when the Reynolds number is held constant. The wall on the inside of the cavity is considerably hotter than the wall on the right, particularly the lower part. When the Richardson number increases, the threshold for critical external heating is reached, and severe temperature gradients appear along the hot wall. Isotherms start to become parallel to the wall at a Richardson number of 100, which indicates that convection is the dominant flow. When Ri = 0.01, the lower half of the hot wall experiences a negative local heat transfer, while the upper half experiences an increase.

When the Richardson number goes up, the amount of local heat that can pass through the hot wall’s bottom area goes up, while the amount that can pass through the tiny portion goes down. The cavity possesses a primary recirculation zone as well as a vortex at the left vertical wall, even when there is no magnetic field present. An increase in the Hartmann number results in an increase in the strength of the magnetic field, which dampens the flow motion. The recirculating zone next to the left vertical wall grows larger as the Hartmann number increases, whereas the other vortex becomes smaller. As the Hartmann number rises, the temperature gradients along the hot wall become more abrupt, while the isotherms become further apart from one another. When the strength of the magnetic field increases, the isotherms become parallel to the cavity walls, which indicates that conduction is the mode of heat transmission. The magnetic field acts to suppress convection, which in turn reduces the amount of heat that is transferred both locally and on average. The increase in heat transmission from Ha = 50 to Ha = 0 is 146.2% better than the previous value.

In a rectangular cavity, researchers [77] investigated the effect that magnetic fields and internal heat generation had on free convection flow. The cavity is stuffed with nanofluid-saturated porous media made of copper and water. The average Nusselt number goes down when the Hartman number goes up, and the solid volume fraction concentration has an effect that is roughly equivalent to that. The average Nusselt number sees a slight increase whenever there is a steeper angle of inclination for the magnetic field. A clockwise spinning cell is created when fluid rises along the source of heat and then flows down the diagonal wall after it has been cooled. As Ha grows from 0 to 10, isotherms and maximum temperatures increase, but for higher values, they are similar. The Lorentz force, which decays fluid motion, grows as Ha does. As the solid volume percentage grows, the maximum streamlines and intensity of the two rotating cells drop due to the cooling mechanism. As the solid volume percentage grows, the maximum temperature drops. In streamlines, the magnetic field inclination angle has a clear influence. The maximum streamlines grow as the magnetic field parameter’s inclination angle increases. The maximum temperature and isotherm intensity increase with magnetic field inclination.

Sheikholeslami [78] investigated the hydrothermal changes that occurred in an alumina–water nanofluid as it travelled through parallel fins inside of a hexagonal enclosure that was only partially heated. Both the bottom and the top of the enclosure are heated, but the top is heated only partially. The fin in the middle is heated, while the right and left fins are cooled. The hexagonal cavity is influenced by the horizontal magnetism. Because of the magnetic effect within the enclosure, there is a secondary circulation near the main circulation zone of the cold fins, while the streamlines near the top of the cold fins appear to move towards the upper wall for both H = 0.3 and H = 0.5. This phenomenon is caused by the fact that there is a secondary circulation near the main circulation zone of the cold fins. It is interesting to note that these upper circulations occur because the intensity of Ha = 50 becomes stronger as it moves up to Ha = 100, but the lower fin’s height has a relatively high intensity. This phenomenon explains why it is possible to observe these upper circulations. When H = 0.7, the size of the main weak circulation increases as well.

Any variation in magnetic strength causes a disruption in their otherwise uniform structure, and the regions with the highest or lowest speed near the tips of the cold fins tend to be on the upper wall. The rate of travel is slowed down when there is a powerful magnetic field present. When there is a magnetic effect, the Lorentz force is physically invited to join the flow regime by the magnetic effect. Since the Lorentz force is slowing, it blocks the convection current, so there is not a lot of distortion or speeding up inside the hexagonal enclosure. In addition, the magnetic field has a strong slowing effect, which makes it seem like the streamlines or speed are moving backwards. Maximum or minimum speed happens in the same area, but the most interesting thing is that the low-speed zones, which look like balloons, are connected at first when there is no magnetic field, but they start to separate along the vertical direction when a magnetic field is applied horizontally. They start to squeeze themselves to obtain a stronger magnetic effect, which makes their speed go down. Because of the way the magnetic field slows them down, they must act in this way. For H = 0.7, there is a clear pattern. For H = 0.3 and H = 0.5, the areas with the highest speeds change at the top boundary and around the heated fin.

The distributions of Ag–water nanofluid inside a square cavity with different temperatures on the vertical and horizontal walls are adiabatic. This is the case when both the magnetic field and the thermal radiation are taken into consideration. The rates of heat transfer slow down as the magnetic field parameter goes up (M). This is because when a magnetic field hits a nanofluid, it creates the Lorentz force, which slows the nanofluid down. Therefore, the values of Nu go down. When a 0.05 volume fraction of silver nanoparticles is mixed with water, the rate of heat transfer goes up from 6.3% to 12.4% [79].

The authors of [80] examined the influence of thermal radiation on the heat transport of magnetohydrodynamic nanofluids in a porous medium. The impacts of the radiation parameter, the CuO volume percentage, and the Hartmann, Darcy, and Rayleigh numbers on hydrothermal behavior are illustrated. It was seen that when nanoparticles are added, the thickness of the thermal boundary layer decreased. Thus, as the volume fraction of nanofluid goes up, the rate of heat transfer goes up. In addition, the nanofluid moves faster than the base fluid because the nanoparticles are moving faster. Furthermore, adding nanoparticles has a bigger effect when there is a magnetic field. When the Darcy and Rayleigh numbers are low, you can see how heat moves, so isotherms look like cylinders. As Ra goes up, the buoyancy forces make the heat transfer through convection even better. Therefore, as Ra goes up, there are more changes in the isotherms, and the maximum goes up as Ra goes up. As the Darcy number goes up, the medium becomes more permeable and the convective mechanism becomes stronger, so as Da goes up, the rate of heat transfer and the absolute values of the stream function go up. As the magnetic field becomes stronger, Lorentz forces are created, and these forces slow down the nanofluid. Moreover, as the Ha goes up, the rate at which heat is transferred goes down.

Researchers [83] have examined the effect of thermal radiation and a magnetic field on the natural convection of heat through a nanofluid within a square cavity. It has been observed that as the magnetic field parameter increases, the size of the vortex contained within the cavity decreases. This is due to the Lorentz force, which restricts the motion of fluid masses and increases in strength as the magnetic field’s strength increases. As M values continue to increase, both the vertex strength and velocity of the vortices in the entire chamber decrease. When the magnetic field’s intensity is increased, the isothermal lines become less curved. Because of this, the mechanism for transferring heat has less of an effect on the whole chamber. Therefore, the mechanism of conduction heat transmission is more prevalent throughout the entire square cavity than convection heat transfer.

Researchers [84] have investigated the effect of radiation and magnetic fields on the convection heat-transfer rate and nanofluid entropy generation in a diagonal square cavity with a conductor fin. It is observed that as the magnetic field strength increases, so does the magnetic field’s Lorentz force. By strengthening the magnetic field, the Lorentz force prevents fluid masses from moving. By reducing the fluid’s motion, the buoyancy force is also diminished. This causes the vortices to move more slowly and diminishes their hold on the fluid. The maximum value of the flow function decreases as the speed and intensity of the vortex that forms in the cavity decrease. This could make heat transfer more difficult.

As the magnetic field strength increases in the temperature field, the number of temperature lines near the walls with constant temperatures decreases. By reducing the density of the isothermal lines, the temperature difference at this location decreases. All modes of heat transfer between locations are slowed when the temperature gradient is reduced. It has also been observed that the curvature of the isothermal lines decreases as the magnetic field strength increases. Reducing the curvature of the temperature lines indicates that the contribution of the heat transfer mechanism in the cavity has diminished while the contribution of the conduction heat transfer mechanism has increased. By strengthening the magnetic field and preventing the Lorentz force it generates, the vortices within the cavity become weaker. Consequently, convection heat transfer decreases.

Researchers [85] studied the effect of radiation and an angled constant magnetic field on the free convective heat transfer and entropy production of an alumina–water nanofluid in a slanted triangular cavity. The maximum heat transfer occurred when the magnetic field angle was 90° and the enclosure angle was 60°. When 6% of nanomaterials were added to water, the heat0transfer rate increased by 7.3% and the total entropy increased by 9.0%. Under the influence of a magnetic field, researchers [89] concentrated on the free convective heat transfer with thermal radiation of an alumina-water nanofluid in an angled cavity. Its shows the ${\mathrm{Nu}}_{\mathrm{avg}}$ on the cavity’s right wall at Ra = $10^{5}$, AR = 0.1, RD = 1, and φ = 0.03, for Ha = 0, 20, 40 and the inclination angle = 0–80.

As is readily apparent, increasing the Ha decreases the heat-transfer rate. This is because increasing the Ha decreases the vortex velocity, which in turn decreases the Lorentz force, which in turn decreases the temperature gradient. When Ha is increased, the mean value of Nu also decreases. In addition, the heat-transfer rate increases when the cavity inclination angle is increased. As the cavity inclination angle increases, the vortex produced in the vicinity of the vortex intensifies, resulting in an increase in velocity in the vicinity of the wall. Consequently, the temperature gradient becomes even more pronounced, which accelerates the rate of heat transfer. When the cavity’s inclination angle is increased, there is less fluid diffusion in the lower portion of the cavity. As a result, heat transfer occurs between the fluid heated by the hot baffle and the cold upper wall, ultimately resulting in a decrease in the heat-transfer rate at the lower wall. By making the cavity more slanted, the rate of heat transfer from the top wall to the bottom wall increases.

Finally, we concluded that future research should focus on other types of nanoparticles, particularly spinel and bio-based (green) nanoparticles, as well as more magnetic and hybrid nanoparticles. Co, Cu, CuO, Al_2_O_3_, Fe_2_O_3_, Ag, TiO_2_, and Cu–Al_2_O_3_–water nanoparticles have previously been used in MHD convection studies (within a cavity). The presence of a magnetic field was found to increase the thermal and convective properties of magnetic nanofluids. However, more extensive experimental research in this area is required. There are many engineering and industrial uses for the effects of non-uniform temperature changes on square cavities. These uses include cooling nuclear reactors, the polymer and metallurgy industries, solar collectors, and other similar uses.

Much research has been performed on using cavities in microchannel heat exchangers. In the next section, we will briefly explain the role of cavities in microchannel heat exchangers. The above review observed that using cavities in nanofluids has an excellent influence on heat transfer and enhances heat transfer.

## 3. Effect of Cavities in Microchannel Heat Exchangers

A device known as a heat exchanger is one that moves heat from one fluid to another, typically from one hot fluid to one cold liquid. The appliance reduces the amount of energy lost and increases the effectiveness of energy use. Heat exchangers transfer thermal energy, also known as heat, between two streams at different temperatures. These devices find widespread usage in industrial processes, either as an integrated part of the process or heat recovery. The energy industry, the power industry, the petroleum industry, the metallurgy industry, and the chemical industry all utilize heat exchangers to recover waste heat. The search for energy sources that are both cost-effective and efficient has resulted in the requirement for heat exchangers that have higher efficiencies at a fair cost. One potential approach is to reduce the size of the heat exchangers to satisfy this need. Improved heat transport will significantly boost heat exchanger efficiency while reducing size and expense. To considerably enhance heat transfer qualities, extensive studies are underway to examine the design of cavities in heat exchangers.

The fluid flow inside and outside the tubes that make up a heat exchanger is a significant factor in determining how well the heat exchangers will do their job. The fact that the fluid is in contact with the heat exchange surface indicates that the heat transfer will occur at the interface surface; as a result, the movement of the nanofluids in these channels is significant for improving the performance of the heat exchangers.

Microchannel heat exchangers are becoming more popular because they are small and can move heat and mass quickly. The pharmaceutical, microelectronic, automotive, air conditioning, solar cell, and refrigeration industries extensively use these heat exchangers [102].

Tuckerman and Pease [103] were the pioneering researchers who proposed microchannel heat exchangers. Researchers [104,105,106,107,108,109,110,111,112,113,114] examined the various cavity forms in microchannel heat exchangers. The cavity increases the heat transfer area, enhances mainstream disturbance, and causes chaotic advection, substantially affecting heat-transfer enhancements. In addition, cavities have shown a small pressure drop cost compared to ribs or fins.

The heat transfer and flow distributions of the microchannel heat sink with various geometric designs of wavy plates were explored statistically by Naphon [115] under conditions of continuous heat flux. According to the findings, the angular edges of the wavy plates greatly impacted the flow structure and contributed significantly to the improvement of heat transfer.

According to previous research, reentrant cavities can impede the formation of the thermal boundary layer within a microchannel heat sink. This behavior interrupts the thickening of the boundary layers and increases the mixing of the flow at the leading edge, increasing heat transfer.

Hou and Chen [106] constructed and tested a microchannel heat exchanger with fan-shaped chambers. The effects of deviation °, coincidence °, and fan-shaped cavity distribution on microchannel heat exchanger performance were studied. Heat exchangers with fan-shaped chambers are more efficient. Spurting and throttling in fan-shaped cavities and interrupted and regenerated thermal and hydraulic boundary layers along the microchannel boost heat transfer. Huang et al. [105] manufactured microchannel heat exchangers with various cavity shapes and tested them experimentally. Compared to a straight channel, the results show that introducing cavities in microchannels helps improve heat transfer while lowering the pressure drop.

We learned that the channel size in heat exchangers varies depending on the application. Reducing the hydraulic diameter is responsible for the enormous heat transfer performance, leading to a significant pressure drop. Furthermore, increasing the channels for the same heat exchanger capacity enhances the heat transfer performance while increasing the pressure drop. Assume that the application of microchannel heat exchangers is for a land-based system where the pressure loss is acceptable. The small size of the microchannel will perform better in this situation to yield a high heat-transfer rate. When evaluating microchannel heat exchangers for space applications, we can increase the channel size to enhance the efficacy because the pressure drop across the system needs to be kept to a minimum to achieve a maximum heat-transfer rate [116].

The different shapes of cavities are square, circular, trapezoidal, and rectangular. If we compare the performance of the other cavities, we know that the circular shape cavities provide the best performance because they provides a high heat-transfer rate with low pumping power. The circular channels are the most efficient at low Reynolds numbers. However, microchannel heat exchangers with triangular channels are more efficient at high Reynolds numbers than trapezoidal and circular channels. This behavior could be explained by the effect of the entry region, as the length of the entrance in triangles and trapezoids is longer than in circular channels and other shapes. The impact of the entrance region increases proportionally with the Reynolds number, the performance loss, and the pressure drop increase. In addition, when Reynolds numbers rise, the performance index falls. As a result, the triangular shape is favored in applications with high Reynolds numbers, Re > 200, whereas in applications with low Reynolds numbers, Re < 200, the circular shape is favored. In general, when Re increases, the efficacy drops due to the increased flow velocity, which reduces the residence time within the microchannel heat exchangers [116].

The shape of microchannel heat exchangers’ reentrant cavities has a significant effect on their pressure drop and heat-transfer performance. Rectangular reentrant cavities had the highest pressure drop and Darcy friction coefficients, indicating they had the best performance in terms of pressure drop. Microchannel heat exchangers with circular reentrant cavities had the lowest hot-water outlet temperature at the same flow rate. In contrast, the rectangular microchannel heat exchangers had the highest temperature. The opposite was true for the temperature of the cold-water outlet. Circular reentrant cavities had the highest Nusselt number. Microchannel heat exchangers with circular reentrant cavities have the highest heat performance [108] in the overall investigation. Research [106] shows how the shape of the cavity affects the pressure drop, how well heat is transferred, and how well the heat exchanger works overall.

Circular reentrant cavities possessed the most significant Nusselt number of microchannel heat exchangers, followed by trapezoidal and rectangular reentrant cavities. This comparison shows that microchannel heat exchangers with circular reentrant cavities produced the most heat, consistent with earlier research indicating that microchannel heat exchangers with circular cavities provide the highest performance. In addition, from the above comparison, in spite of the high performance of circular cavities in micro heat exchangers, the microchannel heat exchangers with square cavities have more applications in the related industries. In order to model a finned tubular heat exchanger, Hejri et al. [117] integrated the numerical results and experimental data. When there is both a temperature difference and gravitational acceleration, a natural convection flow will exist in the heat exchanger. The heat exchanger is filled with a contemporary working fluid, that is, a nanofluid composed of Fe_2_O_3_ and water. Malekshah et al. [118] conducted research on the convective flow that occurs in overheat-dispersing fins. The active fins, which serve as heat sinks, are the primary application of the current problem. This application can be found in the process of cooling an electronic package. In terms of thermal performance, the taller fins are superior to the wider fins in terms of efficiency and effectiveness. Because of the favorable impact that nanofluid has on cooling performance, the use of this substance in electronic packaging for cooling purposes is strongly encouraged.

When an external magnetic field is added to the system, the direction, intensity, and orientation of the magnetic field, as well as the nanoparticle type and fraction, are some of the characteristics that can affect the thermophysical properties of magnetic nanofluids. Because of this effect, an external magnetic field can be used to control flow and heat-transfer processes. The magnetic field effect is applied to the nanofluid moving down the channel, subjecting the nanoparticles to Kelvin forces [119]. As a result, rotational flows form, and the principal flow is directed toward the channel’s wall. As a result, the thermal boundary layer is torn up, resulting in increased heat convection. Lorentz forces, on the other hand, occur in the flow zone depending on the intensity of the applied magnetic field, and Lorentz forces affect both the temperature and the flow profiles. Furthermore, because Lorentz forces act in the opposite direction as the flow, the flow will be distributed in various directions, causing turbulence. As a result, heat transfer leads significantly to an increase in pressure drop.

This section concludes with a summary that the channel size in heat exchangers varies depending on the application. Reducing the hydraulic diameter is responsible for the enormous heat-transfer performance, leading to a significant pressure drop. Furthermore, increasing the channels for the same heat exchanger capacity enhances the heat-transfer performance while increasing the pressure drop. Assume that the application of microchannel heat exchangers is for a land-based system where the pressure loss is acceptable. The small size of the microchannel will perform better in this situation to obtain a high heat-transfer rate. When evaluating microchannel heat exchangers for space applications, we can increase the channel size to enhance the efficacy because the pressure drop across the system needs to keep to a minimum to achieve a maximum heat-transfer rate. Microchannel heat exchangers with circular cavities provide the highest performance. Moreover, from the above comparison, in spite of the high performance of circular cavities in micro heat exchangers, the microchannel heat exchangers with square cavities have more applications in the related industries.

In the next section, we will discuss the effects of geometries on solar collectors for heat transfer enhancement using nanofluid transport.

## 4. Effect of Cavities in Solar Collectors

Global-warming-related climate change has recently come up again. International organizations are still advocating for sustainable energy over conventional fossil fuels. One type of often-used renewable energy is solar energy. Humans have utilized solar energy for a very long time.

A significant problem has been solar energy storage. Researchers have been looking for a viable way to store this time-varying energy for a while now. Geometric modification can improve the efficiency and extraction of solar energy systems for long-term development. Solar collectors require structural changes as well as efficient geometry. Over the last few decades, global warming, ozone layer depletion, and rising sea levels have been just a few of the irreversible environmental consequences of human activity [120,121]. In recent years, many modifications have tried to improve the efficiency of solar systems. There is evidence that both geometrical and fluidic alterations are advantageous [122]. Using nanofluid as an alternative to conventional fluid has proven to be beneficial in collecting more of the solar energy that reaches the earth’s surface [123,124]. A vast amount of research focuses on understanding the overarching goal of expanding solar energy usage and achieving sustainable development. Table 4 details different shapes of cavities used in solar collectors to achieve a maximum heat-transfer rate. The table shows that trapezoidal and triangular cavity geometries are most used in solar collectors and give the maximum heat transfer. It also notes that the solar collector observed complete heat transfer at an angle from 45 to 60°.

Using both theoretical and experimental methods, the current performance of a solar air heater with triangular channels is examined. Using models and several experimental datasets, the influence of the U-turn airflow pattern on the temperature of the air stream as it passes through the collection channels was examined. The thermal output of the heater was evaluated under a variety of operational conditions. A solar air heater with a U-turn airflow pattern and a triangular channel had a 25% smaller surface area than one with a flat plate. The triangle channel air heater had a larger surface area for heat transfer per volume than a flat-plate solar air heater. Moreover, the convective heat transfer coefficient of the airflow within the triangle solar air heater was increased [91]. When the flow is turbulent, air moves from the interior of the duct to the corners on the outside. At corners, it facilitates secondary flow. Secondary flow is described by the components of the velocity vectors along the circular axis (flow is along the z-direction). Even though the secondary flow’s amplitude is much smaller than that of the main flow, it still significantly modifies the characteristics of the turbulent flow. In the cross-section of a triangular duct, the small vortices at the sidewalls of the rectangular duct do not exist. It facilitates a more uniform crossflow between the triangular duct’s sidewalls.

Additionally, the vortex that forms around the triangle duct’s apex, known as the secondary flow, travels inward. Because there are not any smaller vortices near the sidewalls, the turbulent kinetic energy there is higher. Because of this, a smooth triangular-duct solar air heater has a much better heat-transmission rate than a soft rectangular-duct solar air heater [99,100,101]. The turbulent kinetic energy contours show that in the rectangular-duct solar air heater, the intensity of the turbulence kinetic energy diminishes along the sidewalls. The triangular solar air heater contrasts. The rectangular duct had a more prominent boundary layer than the triangular duct, indicating a lower thermal performance [102].

Researchers [99,135] used geometric alteration, nanoparticles, and foam metals to increase the thermal conductivity of a triple-tube latent heat storage system. The aspect ratio affects nanofluids’ ability to generate heat, convection, and entropy. A = 2:1 solar collector nanofluids are typically 93% more efficient than A = 1:1. It provides the effect of aspect ratio in cavities on Nusselt numbers.

Because of nanofluids absorbing heat from the heated wall, the fluid density decreases and nanofluid temperatures rise. The low-density fluid is propelled upward by the density differential. The lower half of the heated wall forms a substantial thermal barrier layer due to the nanofluid’s ability to conduct heat away from it. The boundary layer thickens, reducing heat transfer. Nusselt values decrease with cavity height. As the wall size increases, local Nu decreases. Graphs showing the link between Nu and Ra show that nanofluids with a large Ra will always have a large Nu. Parallelogram and trapezoid cavities are less heat-conductive than rectangle cavities. Heat walls above cavities increase the conduction fraction in heat-transfer mode [99].

At the end of this section, we summarize that using the cavities in solar collectors has very significant results. The cavity’s aspect ratio is critical in designing solar collectors. The internal nanofluid’s ability to generate heat convection and entropy depends on the solar energy collector’s aspect ratio. Compared to A = 1:1, the nanofluids in the solar energy collector with aspect ratio A = 2:1 may typically be enhanced by 93.0%. Moreover, the inclined cavities have good results in the performance of solar collectors; the angle range from 0 to 90 ° gives maximum performance in solar collectors.

Many scholars have recently investigated the use of nanofluids in solar dish collectors because nanofluids can improve thermal performance. This method is very useful in solar dishes, and the higher thermal characteristics of nanofluids make it feasible. Authors [136] investigated the heat transfer of concentrating solar power using a Pt-based nanofluid. They investigated the nanoparticles’ structure. The results show that the thermal conductivity and heat-transfer coefficient of the nanofluids were enhanced by up to 37% and 20%, respectively. They also discovered that using a Pt-based nanofluid improved the thermal performance of concentrating solar power. Aguilar et al. [137] examined the thermal characteristics of a NiO-based nanofluid used as the working fluid of a solar concentrator. They examined the nanofluid thermal characteristics experimentally and discovered that the nanofluid thermal conductivity and heat transfer coefficient improved by up to 96% and 50%, respectively. They suggested using NiO-based nanofluids for the concentrating solar system’s heat transmission. Ref. [138] studied TiO_2_-based nanofluids as the concentrating solar power system’s working fluid. Thermal parameters of the nanofluid, such as isobaric specific heat and thermal conductivity, were considered. They discovered that using the TiO2-based nanofluid instead of the base fluid enhanced the thermal efficiency of the concentrating systems by up to 35%. Ref. [139] investigated the effect of several nanofluids as the heat transfer fluid in concentrating solar power systems. They discovered a variety of nanoparticles, including Cu, Ag, and Ni. The results showed that the thermal properties of nanofluids based on Cu and Ag increased, whereas the thermal characteristics of nanofluids based on Ni decreased.

The use of cavities in solar collectors in nanofluid flow has several advantages. The use of nanofluids has been found to improve thermal performance in all the cavities studied. According to the experimental data, nanofluid use has been proven to be a dependable solution for enhancing thermal efficiency. The average thermal efficiency improvement using nanofluids is 12.90% for the hemispherical cavity, 5.84% for the cubical cavity, and 1.44% for the cylindrical cavity. The thermal efficiency gain is stronger at higher temperatures, making the use of nanofluids a promising option at high temperatures, which are the most relevant cases for solar dish concentrators. Using nanofluid in a cubical receiver improves the thermal performance over using pure thermal oil. The increase in temperature is expected to be 5%. The most efficient cavities are cubical and hemispherical, whereas the cylindrical cavity is the least effective. Although the performance difference between the hemispherical cavity and the cubical cavity is on the small side, it appears to be the better option.

In the literature, several instances of the usefulness of different cavities in multiple applications, such as L-shaped cavities used in the cooling system of nuclear and chemical reactors and electronic components, are examined. Open cavities such as ellipsoidal, triangular, trapezoidal, and hexagonal are applied in electronic equipment cooling, building heating and cooling, and automotive applications. A triangular isosceles cavity has applications in solar thermal collectors. Consequently, cavity design is a crucial part of thermal systems. Appropriate cavity design conserves a substantial amount of energy; hence, suitable cavity design in relevant applications produces favorable rates for heat transfer. The internal nanofluids’ ability to generate heat convection and entropy depends on the solar energy collectors’ aspect ratio. Compared to A = 1:1, the nanofluids in the solar energy collector with aspect ratio A = 2:1 may typically be enhanced by 93.0%.

## 5. Future Recommendations

The above literature review has led to several recommendations for future research efforts. According to the report, the cavities have numerous nanofluid applications for heat-transfer analysis. The following are suggestions for future research

- The internal nanofluids’ ability to generate heat convection and entropy depends on the cavity’s aspect ratio, so it is suggested that more research be focused on using inclined shape cavities in artificial neural networks.
- According to the literature, it is observed that the size of nanoparticles is very important in improving the heat-transfer rate; for smaller sizes, greater heat transfer is attained. After reviewing the many research articles it is recommended that a size of nanoparticles between 10 and 50 nm is more stable in base fluids in cavities at the specified temperature gradient. As a result, the most significant heat transmission may be obtained by making the diameter of the nanoparticles smaller in the cavity. It is suggested that research should focus on different shapes of nanoparticles smaller than 10 nm, with the same design of cavities in microchannel heat exchangers and solar collectors to improve performance.
- In the literature, it is reported that microchannel heat exchangers with circular cavities provide the highest performance. Even though circular cavities provide the best performance in micro heat exchangers, microchannel heat exchangers with square cavities have more applications in related industries, so different shapes of cavities, such as cubical, hexagonal, and conical, should be used in micro heat exchangers in the future.
- In solar collectors, different cavities such as square, rectangular, and triangular cavities are used, and the best results are observed with the use of these cavities, but there is less research focused on conical, hexa-conical, and other novel optimal cavity geometries, so it is recommended that in future research, more focus be given to these types of geometries.

## 6. Conclusions

The present review is a broad perspective on the research progress made in the heat-transfer enhancement and energy conservation of nanofluids using cavities. According to the reviewed literature, cavities in nanofluids have a higher potential for cooling, thermal storage, solar energy components, heat exchangers, and cooling interrelated technologies. Based on the present review, below are the following concluding remarks:

(a) In heat exchangers and solar collectors, the use of a specific shape and design of the cavity provides better results. The shape of the cavities depends on how they will be used, so it is important for thermal systems to have the right cavities.
(b) In the literature, it is reported that L-shaped cavities are used in the cooling systems of nuclear, chemical, and electronic components and give suitable results.
(c) It has been seen that the size of the channels in heat exchangers changes depending on what they are used for. In land-based systems, the smaller the channel, the better the results. This is because the smaller the channel, the smaller the hydraulic diameter, which is what makes the heat transfer work so well. This causes a big drop in pressure. When figuring out how to use microchannel heat exchangers in space, we can make the channels bigger to make them more effective. This is because the pressure drop across the system needs to be kept as low as possible to obtain the best heat-transfer rate.
(d) Different shapes of cavities including square, circular, trapezoidal, rectangular, and others are used in microchannel heat exchangers. From the literature, it is observed that the circular cavities provide the best performance because they provide a high heat-transfer rate with low pumping power and are most efficient at low Reynolds numbers.
(e) The use of nanofluids has been found to improve thermal performance in all the cavities studied. According to the experimental data, nanofluid use has been proven to be a dependable solution for enhancing thermal efficiency. The average thermal efficiency improvement using nanofluids is 12.90% for the hemispherical cavity, 5.84% for the cubical cavity, and 1.44% for the cylindrical cavity.

## Figures and Tables

**Table 1:** Information on different shapes of cavities studied in nanofluid flow.

Ref.	Cavity Geometry	Nanoparticles and Their Size (nm)	Concentration of Nanoparticles	Cavity Inclination Angle	Results
[25]	Square	Fe_3_O_4_, Ha = 0–10	Φ = 0.01–0.04%	-	Heat transfer increases with the increase in Lorentz force effect.
[26]	L-shaped	Ag	Φ = 0.06%	0, 30, 60, 90	The inclination angle has a direct correlation to the amount of heat transferred.
[27]	Isosceles Triangular	Al_2_O_3_, d_p_ = 10 nm, Ha = 0, 25, 50	Φ = 0.06%	$\frac{\pi }{12}$	As Ha and the angle of inclination of the magnetic field went up, the rate of heat transfer went down.
[28]	Closed elbow-shaped	Cu	Φ = 0–0.06%	-	Heat transfer increases due to high nanoparticle volume fraction.
[29]	Trapezoidal	CuO Ha = 0, 10, 50, 100 d_p_ = 29 nm the angle inclination of magnetic field = 0–π	Φ = 0–0.04%	-	The heat transmission rate drops as Ha rises and increases with a high nanoparticle volume fraction.
[30]	Shallow	Al_2_O_3_, d_p_ = 10 nm	Φ = 0–0.04%	-	Radiative heat transfer mixed with natural convection may impact the flow field and cause the rise in Nusselt number (Nu). Thermal radiation research is highly beneficial in the enrichment of heat-transfer rate.
[31]	Wavy-walled	Al_2_O_3_	Φ = 0–0.04%	0–π/2	Inclination angle and undulation number are non-monotonic functions of heat transmission and fluid flow.
[32]	Open wavy	-	-	-	The average Nusselt and Sherwood values can continuously be improved using wavy surface design parameters.
[33]	Porous wavy	-	-	-	Localized heat source affects nanofluid flow and heat transmission rate.
[34]	Vented	CuO, Ha = 0 and 40 d_p_ = 29 nm, the magnetic field inclination angle = 0–π/2	Φ = 0–0.03%	-	In the absence of MHD effect, the nanoparticles increase heat transfer up to 9–9.5%.
[35]	Inclined wavy	CuO, Ha= 0–100, the angle inclination of magnetic field = 0–π	Φ = 0–0.05%	0–π	Changing cavity inclination angle affects convective heat transfer. Heat transmission rate increases with nanoparticle volume fraction.
[36]	Circular	MWCNT-Fe_3_O_4_/H_2_O, Ha = 0–50	Φ = 0–0.03%	-	Convective heat transfer is enhanced by ejecting ${\mathrm{Fe}}_{3}O_{4}-\mathrm{MWCNT}$ hybrid nanoparticles into the host fluid.
[37]	Square	Copper	Φ = 0–0.03%	-	Heat-transfer rate decreases with increasing solid volume fraction for a given Ra, but increases with increasing nanoparticle volume fraction.
[38]	Rectangular	Al_2_O_3_	Φ = 0.0–0.05%	-	Al_2_O_3_/H_2_O nanofluids are more stable than ordinary fluids in a heated rectangular chamber.
[39]	Rectangular	Al_2_O_3_	-	-	Aspect ratio affects heat transfer coefficient and Nusselt number.
[40]	Porous square	Al_2_O_3_, d_p_ = 30 nm	Φ = 0.05–0.4%	-	The porous cavity increases the 10% heat-transfer rate with a 0.05% concentration of nanofluid volume fraction.
[41]	Trapezoidal	Paraffin wax, Graphine	Φ = 0.05	-	Rearranging the direction of the trapezoidal cavity resulted in higher melting.
[42]	Hemispherical	Water-ZnO	-	-	The nanofluid saturated in the porous media improves natural convective heat transfer for the given problem.
[43]	Inclined cube	Al_2_O_3_, TiO_2_, CuO	-	0, 45, 90	Compared to the nanofluids, turbine oil has the maximum Nu anywhere at the inclination angle of the cavity.

**Table 2:** Different parameters investigated in cavities.

Ref.	Numerical Method	Material of Nanoparticles	Range of Ra	Range of Le	Size of Nanoparticles	Nanoparticle Volume Fraction	Inclination Angle of Cavity/Magnetic Field	Range of Pr
[44]	FVM	Al_2_O_3_	$10^{2}\leq \mathrm{Ra}\leq 10^{6}$	$2.62\times 10^{5}\leq \mathrm{Le}\leq 1.05\times 10^{6}$	33 nm	0.01≤φ≤0.04	0°≤θ≤60°	4.623
[45]	FEM	Al_2_O_3_	-	$3.5\times 10^{5}$	33 nm	0.01≤φ≤0.04	-	4.623
[46]	FDM	Al_2_O_3_	$10^{2}\leq \mathrm{Ra}\leq 10^{6}$	$3.5\times 10^{5}$	33 nm	0.01≤φ≤0.04	-	4.623
[47]	FEM	Al_2_O_3_	$10^{3}\leq \mathrm{Ra}\leq 10^{6}$	$3.5\times 10^{5}$	33 nm	0.01≤φ≤0.04	-	4.623
[48]	FDM	-	$10^{3}\leq \mathrm{Ra}\leq 10^{6}$	1000	-	*-*	-	7.0
[49]	FVM	Cu, Al_2_O_3_, TiO_2_	$10^{4}\leq \mathrm{Ra}\leq 10^{7}$	-	$25\;\mathrm{nm}\leq d_{p}\leq 145\;\mathrm{nm}$	0.01≤φ≤0.05	-	4.623
[50]	FEM	Al_2_O_3_	$10^{4}\leq \mathrm{Ra}\leq 10^{7}$	$3.5\times 10^{5}$	33 nm	0.01≤φ≤0.04	-	4.623
[51]	FEM	Al_2_O_3_	$10^{2}\leq \mathrm{Ra}\leq 10^{6}$	$3.5\times 10^{5}$	33 nm	0.01≤φ≤0.04	-	4.623
[52]	FEM	Al_2_O_3_	$10^{3}\leq \mathrm{Ra}\leq 10^{6}$	$3.5\times 10^{5}$	33 nm	0.01≤φ≤0.04	-	4.623
[53]	FDM	Al_2_O_3_	$\mathrm{Ra}=10^{5}$	15,267.8	47 nm	0.01≤φ≤0.05	0°≤θ≤150°	6.51
[54]	FEM	Al_2_O_3_	-	$3.5\times 10^{5}$	33 nm	0.01≤φ≤0.05	-	4.623
[55]	FDM	CuO	$10^{4}\leq \mathrm{Ra}\leq 10^{6}$	9460.61	29 nm	0≤φ≤0.09	-	6.53
[56]	FDM	Al_2_O_3_	$10^{2}\leq \mathrm{Ra}\leq 10^{6}$	$3.5\times 10^{5}$	33 nm	0.01≤φ≤0.04	45°	4.623
[57]	FDM	Al_2_O_3_	$10^{2}\leq \mathrm{Ra}\leq 10^{6}$	$3.5\times 10^{5}$	33 nm	0.01≤φ≤0.04	-	4.623
[58]	FDM	-	$10^{4}\leq \mathrm{Ra}\leq 10^{6}$	1000	-	*-*	-	6.82
[59]	LBM	CuO	$10^{3}\leq \mathrm{Ra}\leq 10^{6}$	-	-	0.01≤φ≤0.05	-	6.2
[60]	FVM	Cu, Al_2_O_3_, TiO_2_	$10^{4}\leq \mathrm{Ra}\leq 10^{7}$	-	$25\;\mathrm{nm}\leq d_{p}\leq 145\;\mathrm{nm}$	0.01≤φ≤0.05	-	-
[61]	FVM	Cu, Al_2_O_3_, TiO_2_	$10^{3}\leq \mathrm{Ra}\leq 10^{7}$	-	$25\;\mathrm{nm}\leq d_{p}\leq 145\;\mathrm{nm}$	0.01≤φ≤0.05	-	-
[62]	FVM	-	-	1≤Le≤10	-	-	0°≤θ≤270°	0.054≤Pr≤10
[63]	SIMULATION	Al_2_O_3_	$10^{7}\leq \mathrm{Ra}\leq 10^{9}$	-	$50\;\mathrm{nm}\leq d_{p}\leq 150\;\mathrm{nm}$	0.01≤φ≤0.03	-	7.0022≤Pr≤7.3593
[64]	Hybrid LBM & TVD	Al_2_O_3_	$10^{3}\leq \mathrm{Ra}\leq 10^{5}$	-	$25\;\mathrm{nm}\leq d_{p}\leq 150\;\mathrm{nm}$	0.01≤φ≤0.04	-	-
[65]	FDM	Carbon Nanotubes	10≤Ra≤10	1≤Le≤10		0.01≤φ≤0.05	-	-
[66]	FVM	CuO, Al_2_O_3_, TiO_2_	-	-	$25\;\mathrm{nm}\leq d_{p}\leq 100\;\mathrm{nm}$	0.01≤φ≤0.04	-	-
[67]	FEM	-	$10^{4}\leq \mathrm{Ra}\leq 10^{6}$	-	-	-	-	6.2
[68]	FEM	-	$10^{3}\leq \mathrm{Ra}\leq 10^{6}$	10≤Le≤100	-	*-*	-	6.2
[69]	FVM	-	30≤Ra≤300	1≤Le≤100	-	*-*	-	-
[70]	FEM	Al_2_O_3_	-	$3.5\times 10^{5}$	33 nm	0.01≤φ≤0.04		4.623
[71]	FEM	-	100≤Ra≤300	1≤Le≤10	-	*-*	-	-
[72]	FVM	Al_2_O_3_, CuO	$10^{2}\leq \mathrm{Ra}\leq 10^{4}$	-	33 nm	0.01≤φ≤0.04	0°≤θ≤60°	10
[73]	FEM	-	100	1000	-	-	-	-
[74]	FEM	-	100	1000	-	-	-	-

**Table 3:** Different parameters studied in MHD nanofluids.

Ref.	Cavities Geometry	Nanoparticles and Their Size (nm)	Hartmann Number	Cavity Inclination Angle	Results
[25]	Square	Fe_3_O_4_	Ha = 0–10		Heat transfer increases with the increase in Lorentz force.
[77]	Finned	Cu	Ha = 0–50	0–90	At 90 degrees highest heat transfer achieved and at 30 degrees lowest heat transfer achieved.
[78]	Porous open	Cu, d_p_ = 29 nm	Ha = 0–60	-	Heat transfer increases with increase in Darcy number.
[79]	Open	Al_2_O_3_	Ha = 0–90	-	$\mathrm{Ra}=10^{4}$ has the largest particle effect at Ha = 30, and for Ra = $10^{5}$ at Ha = 60.
[29]	Trapezoidal	CuO, d_p_ = 29 nm	Ha = 0, 10, 50, 100	0–π	The heat transmission rate drops as Ha rises and increases with high nanoparticle volume fraction.
[80]	U-shaped	Fe_2_O_3_	Ha = 0–30	-	Influence of *n* and Ha on heat transport was studied.
[81]	Irregular cavity	Fe_2_O_3_, d_p_ = 47 nm	Ha = 0–40	0–π/2	Nusselt number rises with inclination angle, falls with Ha.
[82]	Half-annulus	Fe_3_O_4_	Ha = 0, 20, 40, 80	-	Due to Lorentz force from a greater magnetic field, low Eckert and Hartmann numbers decrease the Nusselt number.
[83]	Rectangular	Cu	Ha = 0–60	-	For Ha values between 9 and 12, the heat transmission is not affected by the concentration of nanoparticles.
[34]	Vented	CuO, d_p_ = 29 nm	Ha = 0 and 40	0–π/2	In the absence and presence of a magnetic field, nanoparticles increase heat transmission by 9–9.5%.
[35]	Inclined wavy	CuO	Ha = 0–100	0–π	Changing cavity tilt affects convective heat transmission. Heat transmission rate increases with nanoparticle volume fraction.
[84]	Lid-driven	Cu	Ha = 0–50	0–90	Average heat transmission increases 239.35% at Richardson number 100 vs. 1.
[85]	Curved	Fe_3_O_4_, d_p_ = 47 nm	Ha = 0–60	-	Temperature gradient reduces with enhancement of radiation influence.
[86]	Rectangular	Cu	Ha = 0–100	0–90	The average Nusselt number rises with magnetic field inclination.
[87]	Hexagonal	Al_2_O_3_	Ha = 0–100	-	The analysis shows improved convection, velocity, and thermal results for Rayleigh number, but the opposite for Hartmann number and nanoparticle concentration.
[88]	Square	Ag	-	-	Silver nanoparticles dispersed in water increase heat transfer from 6.3% to 12.4%.
[89]	Porous cavity	Cu, d_p_ = 47 nm	Ha = 0–40	-	Radiation parameter increases heat transport, while Hartmann number decreases it.
[90]	Porous lid-driven	Cu, d_p_ = 45 nm	Ha = 0–40	-	Temperature gradient decreases with Ha and increases with Re.
[91]	Ventilated cube	ZnO	Ha = 100	0, 45, 90, 235	In a magnetic field, ω = 45° offers the best heat-transfer rate, whatever the Reynolds number.
[92]	Square	TiO_2_	-	-	Radiation parameter (R) increases heat transfer from hot wall to cold wall.
[93]	Inclined square	Al_2_O_3_, d_p_ = 47 nm	Ha = 0–40	-	Increasing Rayleigh and decreasing Hartmann increase the heat-transfer rate. For Hartmann number growing from 0 to 40, the Nusselt number drops up to 27%.
[94]	Tilted triangular	Al_2_O_3_, d_p_ = 47 nm	Ha = 0, 20, 40	45	The magnetic field angle does not affect heat transport, entropy generation, or Be. The 90-degree angle had the maximum transfer rate and entropy creation.
[95]	Rectangular	Al_2_O_3_, d_p_ = 47 nm	Ha = 0, 30, 60	0–90	Increasing the magnetic field angle decreases heat transfer and entropy formation and raises Bejan number.
[96]	Trapezoidal	Carbon Nanotube (CNT)	Ha = 0–50	-	Magnetic field effects limited effective convection, although CNT particles increased the average Nu value by 84.3%.
[97]	Inclined	CuO, d_p_ = 29 nm	0–50	0–90	Increasing Hartmann number from 0 to 50 reduces Nusselt number by 32% and 34% for water and nanofluid, respectively.
[98]	Inclined square	Al_2_O_3_, d_p_ = 47 nm	Ha = 0–40	0–90	An increase in Ha lowered heat transport and entropy by 45% and 35%, respectively.
[54]	Double lid-driven square	Al_2_O_3_, d_p_ = 33 nm	Ha = 0–50	45	A rise in Reynolds number or reduction of Hartmann number can increase the heat-transfer rate.
[99]	Wavy	Cu	Ha = 0–50	0–360	Bejan number decreases when Hartmann number, irreversibility distribution ratio, and Richardson number rise.
[100]	Cubic	Cu, Al_2_O_3_, TiO_2_	-	-	The Bejan number decreases with a higher Hartmann number, larger irreversibility ratio, and lower Richardson number.
[101]	Lid-driven	Au, SWCNT, d_p_ of Au= 50 nm d_p_ of SWCNT = 70 nm	Ha = 0–40	-	Nanoparticles and nanofluid velocity affect heat-transfer efficiency.

**Table 4:** Different shapes of cavities used in solar collectors.

Ref.	Cavity Geometry	Results
[125]	Rectangular	At 50° the best performance was achieved.
[126]	Triangular	At 60° the best performance was achieved.
[127]	Triangular pyramid	Inclination improves triangular pyramid solar still by 79.05 percent.
[128]	V-down ribs	Maximum heat-transfer rate is attained at roughness pitch at 45°.
[129]	V-rib triangular	Ribbed triangular duct solar air heater (45°) is superior over various configurations of the ribbed rectangular duct solar air heater at higher mass flow rate.
[130]	Trapezoidal	Thermal stratification in the storage cavity affects energy savings.
[131]	Trapezoidal	Stability deteriorates with the temperature gradient.
[132]	Trapezoidal	The cavity was stable and convective.
[133]	Trapezoidal	Round pipe (multi-tube) receivers absorb more solar radiation than rectangular pipe receivers.
[134]	Circular	Circular geometry and vented absorber plates promote turbulence-induced heat transfer.

## Nomenclature

Ra	Rayleigh number
Be	Bejan number
$K_{r}$	Thermal conductivity ratio of solid wall to pure fluid
v	Velocity
φ	Nanoparticle volume fraction
ρ	Density of the fluid
T	Temperature of the fluid
τ	Stress tensor
$D_{B}$	Brownian diffusion coefficient
$D_{T}$	Thermal diffusion coefficient
p	Pressure
c	Specific heat
k	Thermal conductivity
$\rho_{p}$	Density of the nanoparticles
$c_{p}$	Nanoparticle specific heat
t	Time
μ	Dynamic viscosity
$d_{p}$	Nanoparticle diameter
β	Volumetric thermal expansion coefficient
$k_{B}$	Boltzmann’s constant
$\nabla^{2}$	Laplacian operator
y	Rectangular coordinate
$\varepsilon_{p}$	Momentum eddy diffusivity
$\varepsilon_{M}$	Energy eddy diffusivity
$\varepsilon_{H}$	Particle eddy diffusivity
f	Friction factor
Re	Reynolds number
Pr	Prandtl number
$\delta_{v}$	Laminar sublayer
Nu	Nusselt number
${\mathrm{Nu}}_{\mathrm{tot}}^{-}$	Average total Nusselt number
D	Solid block
Ri	Richardson number
Ha	Hartmann number
R	Radius of the cylinder
Np	Nanoparticle
Le	Lewis number
